# Prevalence and causes of vision impairment in East Africa: A narrative review

**DOI:** 10.4102/jphia.v16i1.1273

**Published:** 2025-07-31

**Authors:** Benedict Ayobi, Rekha Hansraj, Nishanee Rampersad, Gerard Urimubenshi

**Affiliations:** 1 Discipline of Optometry, Faculty of Health Sciences, University of KwaZulu-Natal, Durban, South Africa; 2 Department of Ophthalmology, College of Medicine and Health Sciences, University of Rwanda, Kigali, Rwanda; 3 Department of Physiotherapy, College of Medicine and Health Sciences, University of Rwanda, Kigali, Rwanda

**Keywords:** vision impairment, blindness, refractive error, cataracts, East Africa

## Abstract

**Background:**

Vision impairment (VI) affects the quality of life of individuals; it negatively impacts education, mobility and socioeconomic life, leading to dependency. Increased life expectancy is expected to drive a corresponding rise in the prevalence of VI. Timely and effective efforts are required to reduce the burden of VI. Accurate and up-to-date data regarding the prevalence and causes of VI are essential for practical planning to address its challenges and impact. This review therefore presents the prevalence of VI in East Africa.

**Aim:**

This review sought to report the prevalence of VI in East Africa.

**Setting:**

The study was conducted using findings of studies on VI from East Africa.

**Method:**

A search of published literature was conducted using online databases including PubMed, Ovid, Science Direct, Google Scholar, Embase and Medline. The search was restricted to sources published in English and in peer-reviewed journals from January 2010 to November 2023. Only studies that stated the prevalence and causes of VI among the general population during the period in question were included.

**Results:**

Twenty nine studies met the inclusion criteria and were included in this review. The prevalence of VI reported in East Africa by the studies reviewed ranged from 1.6% to 42.1%.

**Conclusion:**

The reported prevalence of VI in East Africa is higher than that reported in other regions of Africa and globally.

**Contribution:**

The review highlights the need for adequate strategies and support to be channelled towards making eye care accessible and affordable in East Africa including the training of more eye health personnel.

## Introduction

The sense of vision is one of the most important senses for human existence; hence, any vision impairment (VI) is expected to negatively impact the quality of life.^1^ According to the International Classification of Diseases (ICD-11):^2^

VI is defined as presenting distance visual acuity (PVA) less than 6/12 but better or equal to 3/60 and/or a corresponding visual field (VF) of less than 20 degrees around central fixation in the better eye, with presenting optical correction, if any. (n.p.)

Globally, it is estimated that at least 2.2 billion people have VI, and in about half of this number, VI could have been prevented or is yet to be addressed.^3^ According to the Vision Loss Expert Group study, as of 2020, the global prevalence of VI was 4.34%. They further estimated that there are 43.3 million people who are blind and an additional 295 million people living with moderate to severe VI out of a total world population of 7.79 billion.^4^ The primary causes of global VI have been identified as uncorrected refractive errors (URE), cataracts, glaucoma, age-related macular degeneration, myopic macular degeneration and diabetic retinopathy.^3,4^

The socioeconomic impact of VI is numerous and affects persons of all age groups. In children, poor vision and the inability to read material written on the blackboard can profoundly impact their participation and learning in class, adversely affecting their education and consequently impacting their future occupation and socioeconomic status.^5,6^ In other age groups, the impact of VI includes ‘loss of educational and employment opportunities, loss of economic gain for individuals, families and societies, and impaired quality of life’.^7^ Additionally, tasks that require ambulation, particularly in challenging environments which rely on peripheral vision, are adversely affected by VI.^5^ It is also established that VI increases the risk of mortality. People with moderate VI have a 57% higher risk of dying earlier compared with those without VI.^8^

Current literature reveals an unequal distribution of the prevalence of VI across the globe. Regarding regional distribution, the prevalence of VI in developing countries (low- and middle-income countries) including East Africa (Burundi, Comoros, Djibouti, Ethiopia, Eritrea, Kenya, Rwanda, Seychelles, Somalia, South Sudan, Sudan, Tanzania and Uganda) is estimated to be four times higher than in high-income or developed regions.^9^ The major contributors to this disparity include limited access to eye care services, lower socioeconomic status and/or lack of data from developing countries.^7,10^ According to the World Health Organization (WHO), most cases of VI are preventable or treatable, yet they remain unaddressed because of lack of access to eye care services, unavailability of suitably qualified eye personnel, cost, stigmatisation and discrimination.^3,11^ Interventions to ascertain the prevalence and burden of disease by early diagnosis, documentation and treatment of conditions that cause VI will help in the planning and implementation of strategies to curb the prevalence of VI. Most studies on VI, especially in Africa, have focused on subgroupings of the population. To curb the VI menace, a comprehensive picture of VI is essential. Thus, the purpose of this review is to document the prevalence, causes and classifications of VI in the general population in East Africa, which is regarded as the fastest growing region on the continent.^12,13^ The results from this study will be baseline data for planning and monitoring VI intervention programmes in East Africa.

## Methods

A preliminary search was conducted in January 2024 to identify relevant literature and validate the research topic, ensuring that it was not previously undertaken. The principal researcher searched published literature using online databases, including PubMed, Medline, Ovid, Google Scholar, Science Direct and Embase. The identified studies were cross-checked by the co-authors. The keywords *vision impairment* (*VI*), *prevalence of vision impairment, causes of vision impairment,* and *East Africa* were used. Other keywords from the retrieved studies were also searched to improve the data. The search was restricted to primary sources published in English and peer-reviewed journals from January 2010 to November 2023. Only studies that reported on the prevalence and causes of VI among the general population for the period in question were included. Abstract-only papers, unrelated articles, studies outside the study settings, the study period, and grey literature were excluded. The study period was limited from January 2010 to November 2023 to minimise the effect of classification differences. Studies published earlier than January 2010 and available from the subregion used ICD-10, while newer studies utilised ICD-11.

This narrative review thereafter synthesised existing research findings on VI in East Africa by comprehensively summarising each relevant study within the outlined criteria. The studies were critically evaluated to compare and contrast methodologies, findings and interpretations. Particular areas of focus included study designs, sample size, recruitment strategies, prevalence and causes of VI and diagnostic tools and measurement techniques employed.

### Ethical considerations

Ethical clearance to conduct this study was obtained from the University of KwaZulu-Natal Humanities and Social Sciences Research Ethics Committee (HSSREC) (No. HSSRED/00006477/2023).

## Results

### Prevalence of vision impairment in East Africa

Twenty-nine studies met the inclusion criteria and were included in this review, including six Rapid Assessment of Avoidable Blindness (RAAB) studies, seven school-based cross-sectional studies, seven hospital-based studies and nine community-based cross-sectional studies. The prevalence of VI reported in East Africa by the studies reviewed ranged from 1.6% to 42.1% (Online Appendix 1 Table 1). Additionally, the causes of VI identified by the various studies have been elaborated in Online Appendix 1 Table 1.

### Prevalence of vision impairment in the different countries in East Africa

Following the literature search, it was noted that there was a dearth of research from East Africa that reported on the prevalence of VI. Of the 13 countries that constitute East Africa,^13^ prevalence data on VI were available for only eight countries and are detailed below. In the period between 2010 and 2023, 11 of the 29 studies identified reported on the prevalence of VI in Ethiopia. This was followed by five studies from the Republic of Uganda. The Republic of Rwanda and Kenya reported four studies each during the specified period. Two studies were published from Tanzania, and one each was published from the Republic of Burundi and Eritrea. There were no prevalence studies noted in Djibouti, Seychelles, Comoros Island, Somalia or South Sudan in the period under review.

#### Burundi

In Burundi, only one study on this subject was conducted, and this was undertaken over a decade ago. It entailed an RAAB study carried out in 2012 among participants aged 50 years and older. Out of 3684 participants, a prevalence of VI of 1.7% was found based on presenting VA (PVA).^14^ This was relatively lower compared to other RAAB studies in East Africa, specifically Rwanda (4.4% and 5.3%)^15,16^ Kandeke et al. suggested that the low prevalence could be because of positive health-seeking behaviour among Burundians and increased efforts by the government. They also speculated that the vulnerable, especially people with blindness and VI, may have succumbed to the conflicts that happened in Burundi in the past.^14^ More women were reported to have severe VI than men (0.7% compared to 0.5% for men). However, a higher prevalence of moderate VI was reported in men (2% compared to 1.4% in women).

#### Eritrea

An RAAB study conducted in Eritrea, a smaller East African country, sampled a total of 3163 participants who were 50 years and older, with 41.9% being male. The reported prevalence of moderate VI (PVA less than 6/18 but better than or equal to 6/60) was 10.5%, while the prevalence of severe VI (PVA less than 6/60 but better than or equal to 3/60) was 3%. In terms of gender, more men than women had VI. For example, 13.7% of men had moderate VI (PVA < 6/18 but better than or equal to 6/60), while 10.8% of women had moderate VI. Furthermore, 4.1% of men had severe VI compared with 2.7% of women.^17^

#### Ethiopia

Eight of the 11 studies that reported on VI in Ethiopia were carried out on paediatric populations and reported a prevalence of VI between 1.8 and 11.7%. Notably, the age classification for the paediatric population differed across the studies. While some researchers limited their definition of the paediatric population to school-going children (5–18 years) as defined by the United Nations (UN),^18^ this review found that the age ranges considered included 5 years to 15 years,^19^ 5 years to 16 years,^20^ 7 years to 17 years,^21^ 11 years to 16 years^22^ and 8 years to 18 years.^23^ Asferaw^24^ was relatively more versatile in his classification of a paediatric population as 6 years to 25 years. Comparatively higher prevalence of VI in adult populations (> 18 years) was reported by the remaining three studies, which ranged between 16.8% and 36.5%.^25,26,27^ All studies based the diagnosis of VI on PVA. None of the studies in adult populations in Ethiopia reported on the prevalence of VI stratified by gender. In the paediatric populations, a higher prevalence of VI was reported in males (52% males compared to 48% females)^22^ (52.9% males compared to 47.1% females).^23^

#### Kenya

Two cross-sectional studies were carried out among paediatric populations in Kenya. Barasa et al.^28^ reported that the prevalence of VI among 5- to 22-year-olds was 4.77%, and this is almost double that reported by Muma and Obonyo^29^ of 2.4% in a slightly younger population (5- to 16-year-olds). The study by Barasa et al. was conducted in an economically less endowed location with limited access to healthcare. The authors speculated that this may have accounted for the higher prevalence of VI noted. In both studies, the prevalence of VI was noted to be higher in females than in males.^28,29^ A higher prevalence of VI was recorded in the adult population, which could be as a result of a comparatively stricter visual acuity (VA) threshold for the diagnosis of VI, that is VA poorer than 6/12.^30,31^ Mathenge et al. reported the prevalence of mild, moderate and severe vision impairment (MMSVI) among persons 50 years and older to be 5.1%, 8.1% and 0.4%, respectively.^30^ Similarly, Signes-Soler et al. reported the prevalence of VI based on PVA to be 6.3% in an adult population older than 18 years of age.^30^ In terms of gender, Mathenge et al. reported a higher prevalence of MSVI in men than in women (8.3% and 0.5% in men compared to 7.9% and 0.3% in women, respectively); however, a higher prevalence of mild VI was reported in women (5.2% compared to 5.0% in men).^30^

#### Rwanda

Among adults, an RAAB study was conducted in the western province of Rwanda involving 2206 participants aged 50 years and above, with the prevalence of VI reported as 5.3%.^16^ A similar RAAB study involving all provinces of Rwanda, also by Mathenge et al., almost a decade later, among 5065 participants aged 50 years and above, reported a relatively reduced prevalence of VI of 4.4% and blindness of 1.1%.^15^ The reduction in VI, according to the study, was because of the increased efforts by the government and donor partners in tackling VI in the country. Lower values were also noted in the studies in younger populations. A prevalence of 1.6% was noted in children 7 years and younger,^32^ while more recently, El-Khoury et al. in a cross-sectional retrospective hospital-based study, reported the prevalence of severe VI in a sample younger than 18 years to be 4.2%.^33^ In terms of gender, Mathenge et al.^15^ reported that more women than men had moderate VI (5.8% of women compared with 4.7% of men); however, the prevalence of severe VI was higher in men (1.5% of men compared with 1.1% of women). Almost a decade later, the gender trend of VI had not changed, although with reduced prevalence values. The prevalence of moderate VI was 2.1% among women and 2.0% among men. The prevalence of severe VI was 0.7% for both men and women. However, when all cases of VI were considered, the prevalence of VI was marginally higher in women (4.8%) than in men (4.0%).^16^

#### Sudan

Alrasheed et al. conducted the only study in Sudan, and reported the prevalence of VI in a paediatric population (6–15 years) to be 4.4%, 6.4% and 1.3%, respectively, based on PVA, uncorrected VA and best-corrected VA, in a school-based cross-sectional study among 1678 pupils. The study revealed that there was no statistically significant difference in the prevalence of VI based on gender.^34^

#### Tanzania

In the period under review, two studies were published from Tanzania on VI: a community-based cross-sectional study^35^ and an RAAB study.^36^ Mashayo et al. reported the prevalence of VI in the general population (over 15 years of age) to be 10.4%,^35^ while Habiyakire et al. reported the prevalence of VI in people 50 years and older to be 5.4%.^36^ The significant difference between the prevalence reported by the two studies could be attributed to the age profiles of the study populations involved in each study. While Habiyakire et al.^36^ reported a lower prevalence, they did not account for persons younger than 50 years. Also, according to Mashayo et al.,^35^ the study was conducted in a ‘hard-to-reach’ district with less access to eye care. Habiyakire et al. reported almost similar estimates of VI between men and women (2.7% men versus 2.2% women).

#### Uganda

In a cross-sectional study among 318 paediatric participants (< 18 years of age), and based in a children’s referral hospital in Uganda, Kinengyere et al. found a prevalence of VI of 42.1%.^37^ All other relevant studies in Uganda were on adult populations and reported the prevalence of VI to range between 6.1% and 32.1%.^38,39,40,41^ Generally, the prevalence of VI reported in Uganda is higher compared with the other East African countries. Furthermore, the aetiology of the study populations may be a contributing factor, as all prevalence studies in Uganda were hospital-based. This may limit the variety in the sample, as most people presenting to hospitals are usually aware they are visually impaired and in need of health care.

#### Causes of vision impairment in East Africa

In the period under review, the major causes of VI reported in East Africa were URE, cataracts, glaucoma, corneal opacities, retinal diseases (including diabetic retinopathy, hypertensive retinopathy, age-related macular degeneration and macular holes) and amblyopia. Other causes of VI included nystagmus, cortical blindness, vitamin A deficiency and hereditary diseases.^14,15,16,17,19,20,21,22,23,24,25,26,27,28,29,30,31,32,33,34,35,36,37,38,39,40,41,42,43^ Uncorrected refractive errors accounted for between 1.7%^24^ and 94%^21^ of cases in this narrative review. Cataracts contributed between 2.7%^43^ and 54.8%^36^ of cases, while corneal opacity accounted for between 0.6%^31^ and 65%^24^ of VI in East Africa.

In the paediatric populations included in the studies in East Africa, the major causes of VI reported were URE, amblyopia and cataracts. The prevalence of URE as the cause of VI in paediatric populations varied from as low as 1.7%^24^ to 94%.^21^ Among those with VI in East Africa, the prevalence of amblyopia ranged from 2.2%^24^ to 22%^29^ and the prevalence of cataract reported ranged between 2.7%^43^ and 18%.^33^ In contrast, the underlying conditions that contributed to the prevalence of VI in the adult populations in East Africa were untreated cataracts, glaucoma, URE and retinal diseases. The prevalence of cataracts in the adult population in East Africa with VI ranged from 8.1%^31^ to 54.8%.^36^ Glaucoma contributed between 0.9%^15,31^ and 36.8%,^26^ while the prevalence of uncorrected refractive error accounted for 7.95%^26^ to 67%^14^ of VI in adults across East Africa.

## Discussion

### Implications and recommendations

Of the 13 countries that constitute East Africa, data on the prevalence of VI were found for only eight countries in the period of the review. There was no published data for Comoros, Seychelles, Djibouti, South Sudan or Somalia. Possible reasons for the lack of prevalence data in the aforementioned countries could include a lack of funding for research purposes, among others.^44^ Additionally, unavailability or lack of interest among eye care personnel to conduct research studies documenting the prevalence and causes of VI may have contributed to this. To better illustrate the latter point, Somalia has only 12 ophthalmologists serving a population of 15 million people.^45^ Such deficiencies may increase the workload of the qualified staff and reduce their availability to engage in research studies. Therefore, there is an urgent need to increase the number of qualified personnel in East Africa to tackle the prevalence of VI. Increased governmental scholarship and post-training bonding of the eye care personnel (ophthalmologists, optometrists and ophthalmic nurses) in serving their communities will contribute significantly to increasing access to eye care services and also reduce patient-doctor ratios in the region.

#### Prevalence of vision impairment in East Africa

Overall, the prevalence of VI in East Africa ranged from 1.6%^32^ to 42.1%^37^ during the chosen study period. Compared to the crude global prevalence range of mild VI (0.88% – 4.77%) and moderate to severe VI (1.34% – 4.89%),^4^ and a similar prevalence of MSVI reported by Naidoo et al.^46^ of 4.0% in sub-Saharan Africa in 2010, the prevalence of VI in East Africa had a relatively wider range (1.6% – 42.1%) and was higher. These differences could be because of the limited studies from East Africa in the period under review and varying criteria used by the studies included in the review. In a systematic review, Bourne et al.^4^ reviewed 512 studies from 98 countries (from January 1980 to October 2018), while the current review studied 29 studies from only eight countries and for a shorter review period. Similarly, Naidoo et al.^46^ conducted a systematic review of 252 population-based studies from sub-Saharan Africa from January 1990 to January 2012. Furthermore, of the 29 studies in East Africa in the current review, 18 were published from 2017 onwards, compared with the earlier studies included in the reviews by Bourne et al. and Naidoo et al. The review by Naidoo et al. used stricter inclusion criteria by only including population-based studies. Studies that used hospital-based data, blindness registries, and self-reported vision status, which may create a bias when estimating prevalence, were excluded from their review. In their systematic review, Bourne et al.^4^ included only population-representative studies. The current review was more versatile and included all studies on VI published in East Africa in the given period. Hospital-based studies and some population-based studies have an inherent limitation in that participants are usually people with known VI seeking medical attention. This was noted in this review, where the hospital-based studies that reported the prevalence of VI from Uganda noted a higher prevalence of VI. Additionally, some population-based studies, which are not population-representative, usually carried out as a community outreach on a walk-in basis, will report a higher prevalence of VI.

#### Criteria for diagnosing vision impairment

A significant aspect of VI is visual field loss, as defined by the ICD-11. However, all the studies reviewed reported on the prevalence of VI based solely on VA, without reflection on visual field (VF). Thus, reporting on the prevalence of VI based on VA only may not represent a comprehensive picture of the prevalence of VI. The seeming neglect of consideration of VF by all studies in reporting VI could be linked to logistical constraints. Automated VF tests (VFT) (perimetry) are usually bulky and require electricity, making them cumbersome for use in mobile clinics. Additionally, it is comparatively more time-consuming than recording VA, which reduces the feasibility of the study. Furthermore, visual field loss is usually secondary to other primary conditions such as glaucoma; most researchers therefore rely on the primary diagnosis, namely, VA measurements, which are relatively less cumbersome to identify and enumerate. We recommend the inclusion of confrontational VFT (CVFT) in the definition and classification of VI. The CVFT, even though a gross test, will contribute to a better understanding of the extent of VI. Automated confrontation testing devices, which are portable and have higher sensitivity, can be considered for hard-to-reach areas without access to standard VFTs.^47,48^ It is simple, easy to administer and can be administered with little training.

#### Causes of vision impairment

Globally, the leading causes of VI have been identified as URE, cataracts, glaucoma, age-related macular degeneration, myopic macular degeneration and diabetic retinopathy.^3,4,49^ These findings are supported by this narrative review, where URE, untreated cataracts and corneal opacity were also noted to be the major causes of VI reported in those with VI in East Africa. Alrasheed et al. suggest that the increased use of technology (urbanisation) and being indoors may be contributing to the rise in the prevalence of refractive error, especially in children.^34^ Bourne et al., agree with that assertion and add that an increasing population and an ageing population have increased the need for eye care services, which have been insufficient to cater for the rising demand.^4^ Therefore, URE as the second leading cause of VI in adult populations may be a spillover from unmet demands in childhood populations. The finding of cataracts and corneal opacities as major causes of VI in East Africa is also similar to that noted by Naidoo et al. in sub-Saharan Africa, which may relate to certain conditions like trachoma being endemic in the continent, as well as limited access to health care, including cataract surgery, in developing countries.^46,50^

#### Causes of vision impairment in various age groups

One noteworthy observation from this review is the higher prevalence of VI noted in paediatric populations, usually driven by a higher prevalence of URE. The reason that may account for the difference in the causes of VI between paediatric and adult populations could be summarised as follows: children may have limited awareness of poor vision, coupled with less access to eye care, consequently, most cases of URE will go unnoticed in children because they are unaware that their vision is impaired.^51^ On the other hand, cataracts and retinal diseases are most prevalent in the adult population, probably because of metabolic changes that occur in the body as a result of ageing.^52^

#### Treatment of the causes of vision impairment

Of the causes of VI reported, the two most prevalent underlying conditions, URE and cataracts, are treatable.^4,15^ Uncorrected refractive error can be diagnosed efficiently and often treated by dispensing spectacles or contact lenses or via refractive surgery. Cataracts can be managed surgically. These simple, yet effective treatment interventions offer great opportunities to reduce the global burden of VI. However, access to and affordability of these interventions often pose the greatest challenge, particularly in developing countries like those in East Africa^4^. Thus, increased vision screening to identify URE and treatment using spectacles are necessary to stem VI. Additionally, improved cataract surgical rates (CSR) are important to address the reversible prevalence of cataracts, with alleviation campaigns such as community outreaches and eye camps to tackle untreated cataracts in East Africa.

#### Distribution of vision impairment according to gender

Analysis of VI trends based on gender in this review revealed that VI is generally noted to be more common in women than in men.^15,19,37,42^ This finding is consistent with that reported by Naidoo et al. in sub-Saharan Africa (3.8% in men [3.1% – 4.7%] and 4.2% for women [3.6% – 5.3%]).^46^ The reasons attributed to this include: women live longer than men and therefore have a higher risk of age-related diseases than men; inequalities in accessing health care, as men have a higher capacity to access health care than women; or a general reflection of global demography, where there are more women than men in the world.^9,44,53^ However, two studies in the current review reported contrary results with a comparatively higher prevalence of VI in men than in women, which may be attributed to the demography of the study populations involved in those studies.^20,23^ Both studies were carried out in basic schools in Ethiopia, and a basic school enrolment report revealed more men than women enrolled in schools in Ethiopia in the period under review.^54^

### Strengths and limitations

This review identified the prevalence and primary causes of VI in the East African region. It has highlighted the distribution of VI and the discrepancies in VI reporting criteria in the subregion. This is the first study to have done this. Aside from the paucity of research from some countries in East Africa, most of the studies in this review had large sample sizes (312–5065) that could justify the generalisation of the findings to other countries with similar demographics in East Africa. A significant limitation of this study was the dearth of research from East African countries in the period under review. Additionally, differences in the criteria used to report on and classify the prevalence of VI limited the quantitative comparisons across studies.

### Recommendations

Early identification and treatment of the treatable causes of VI to alleviate the burden of VI on the general community is a feasible approach to reduce the prevalence of VI.^7,23,34^ To this end, adequate strategies and support should be channelled towards making eye care accessible and affordable. Strategies such as the provision of medical insurance and the provision of tax incentives for health facilities that are set up in rural areas will improve access to healthcare and encourage investment in underserved areas. Furthermore, regular interventions (such as eye camps) that increase awareness of eye health and ensure adequate supply of ophthalmic services need to be encouraged.^55^ Additionally, governments in East Africa should invest more in the training of eye care personnel. A uniform reporting guideline for VI will also be required to guide future studies.

## Conclusion

This narrative review provides an overall view of the prevalence of VI across East Africa and is the first paper to do so. It shows that the prevalence of VI varies across the studies and countries. The reported prevalence of VI in East Africa is higher than that reported in other regions of Africa and globally. The variations may be because of the differences in the diagnostic criteria used to identify VI, sampling techniques and research methodologies used in the various studies. Other factors that may have accounted for these differences include varying access to eye care services in the study populations, socioeconomic disparities in the communities and lack of awareness of good vision, availability, accessibility and affordability of eye care services, as well as the availability of suitably trained practitioners to provide these services and gather epidemiological evidence. Furthermore, this review identified that the leading causes of VI in East Africa are treatable.
